# Premature Ovarian Failure: A Critical Condition in The Reproductive Potential with Various Genetic Causes

**Published:** 2014-03-09

**Authors:** Farkhondeh Pouresmaeili, Zahra Fazeli

**Affiliations:** 1 Department of Medical Genetics, Faculty of Medicine, Shahid Beheshti University of Medical Sciences, Tehran, Iran; 2 Infertility and Reproductive Health Research Center (IRHRC), Shahid Beheshti University of Medical Sciences, Tehran, Iran

**Keywords:** Premature Ovarian Failure, Infertility, Amenorrhea

## Abstract

Premature ovarian failure (POF) is identified as a heterogeneous disorder leading to amenorrhea and ovarian failure before the age of 40 years. The first known symptom of the disease is having irregular menstrual periods. The phenotype appearance of POF depends significantly on the variations in hormones. Low levels of gonadal hormones (estrogens and inhibins) and increased level of gonadotropins [luteinizing hormone (LH) and Follicle stimulating hormone (FSH)] (hypergonadotropic amenorrhea) are well documented as causes of POF. There is an association between the failure of germ cell development and complete ovarian failure, and consistently decreased number of germ cells is more likely associated with partial ovarian failure resulting in secondary amenorrhea. A literature review on recent findings about POF and its association with genomic alterations in terms of genes and chromosomes. POF is a complex heterogeneous disorder. Some of POF cases are carriers of a single gene mutation inherited in an autosomal or X-linked manner while a number of patients suffer from a chromosome abnormality like Turner syndrome in mosaic form and manifest secondary amenorrhea associated with ovarian dysgenesis. Among many of the known involved genes in POF development, several are prove to be positively associated to the disease development in different populations. While there is a promising association between X chromosome anomalies and specific gene mutations with POF, genome-wide analysis could prove a powerful tool for identifying the most important candidate genes that influence POF manifestation.

## Introduction

Premature ovarian failure (POF [MIM 311360]) is an early ovarian malfunction different from menopause, which disturbs production of follicles resulting in amenorrhea under the age of 40 in 1-3% of reproductive age women (1-3). About 10-28% of the patients experience primary amenorrhea and about 4-18% show secondary Amenorrhea (2). Affected women show menstrual problems followed by an elevated level of gonadotropines [follicle stimulating hormone (FSH) ≥40IU/L] and hypoestrogenism for an average four months (4). Measuring serum FSH is a routine diagnosis procedure for the disease (2, 5). Genetic analysis of the early menopause patients showed that a variety of gene defects and chromosome anomalies involving X chromosome and autosomes were associated with POF. The studies confirmed that 

the multifactorial and heterogeneous biological events including infection, autoimmune disorders and metabolic factors are likely responsible for the disorder development. In 90% of observed cases, the etiology is unknown and the disease is defined as idiopathic POF (2, 6-8).

Most POF cases are sporadic and it is suggested that between 4-31% of them are familial (9-11). In this regard, the abnormalities of the X chromosome are presented as the most important causes of the disease (12-17), followed by the fragile X mental retardation (FMR1) premutation which is present in POF patients with frequencies of 13 and 6%, respectively (18, 19).

Loss of one X chromosome as X monosomy [Turner syndrome (TS)], the related gene deletions and X/autosome translocations, trisomy X, X linked gene mutations and premutations and anomalies of autosomal linked genes have been widely studied in correlation with POF disease. In examining the genetic mutations responsible for POF, each mutation could affect part of the disease phenotype. The diagnosis of the disease could be confirmed by two separate blood tests for FSH (4). Studies in different populations have shown various factors in association with POF. In addition to the genetic anomalies and chromatin structure of specific genome environment, autoimmune factors and toxins are reported as other important causes of the disease. The exact reason for POF development still remains unknown in many cases.

### Cytogenetic analysis for POF

#### Chromosome abnormalities

 In different reports, chromosomal abnormalities have been recognized as the most common causes of POF disease (12-17, 20), confirming the importance of cytogenetic analysis in reproductive management and genetic counseling for this disease. Investigations in this regard show the association of POF with chromosome abnormalities, particularly those of X chromosome such as structural anomalies, translocation of X with autosomes, isochromosomes and the related aneuploidies (21-25). Also, translocation of Y chromosome heterochromatic regions on derivative X chromosome which affected the X chromosome inactivation was reported (26). Presence of a Y chromosome in a woman’s genome is a clear sign of chromosome abnormality which mostly causes tumor formation in mosaic karyotypes. Pouresmaeili et al. reported a patient with POF who carried aneuploidy of this kind (27). Unknown X-linked gene imbalance is an expected cause of POF in these patients.

A critical region from Xq13.3 to Xq27 has been characterized for ovarian development and function (28). Studies have shown that deletion of the short arm and the long arm of the X chromosome result in either early primary or secondary amenorrhea (29). These observations suggested that important genes for normal ovarian function are located on both arms of the X chromosome (30).

Translocational studies between X chromosome and autosomes have been significant in determining the involvement of autosomal as well as X chromosomal genes in the development of POF disease. For example, the association of HS6ST1, HS6ST2, MATER and CHM genes with POF were identified following to the analysis of the POF patients with karyotypes 46, X, der (X) t (X; 19) (p21; q13), 46, X, t (X; 2) (q21; q14), 46, X, der (X)t (X; Y) (q25-26; q11.22), 46, X, t (X; 4) (q21.2; p16.3) respectively (31, 32). One possible explanation for the disease occurrence in these cases was attributed to the positional effect of an autosome- X chromosome translocation. In fact, transferred genes to the highly heterochromatic region of the X chromosome tolerate epigenetic effects after a rearrangement which changes their chromatin structure and consequently result in lower expression of genes associated with ovarian function and fertility (33).

Moreover, some studies have reported a Robertsonian translocation (13, 14) in some women with sporadic POF. It was also suggested that the functional changes and interruptions in some critical genes for ovarian function on acrocentric chromosomes, due to translocation, could be a possible reason of the disease etiology in this group of patients (20, 34, 35).

Also, there are POF patients with trisomy X who were diagnosed after showing an endocrine disorder, hypergonadotropic hypogonadism (36). It is suggested that the genes located on the X chromosome escaping inactivation could be overexpressed in 47, XXX patients, resulting in the disorder revelation (37). It is identified that complete 

absence or a segmental deletion of one X chromosome (TS) causes abnormalities throughout the reproductive system. Using fluorescent in situ hybridization (FISH) and several specific markers to the short arm of X chromosome including DXS1058, DXS6810, DXS1302 and ZXDB, deletion of Xp11.2-p22.1 was introduced as a critical area related to TS and POF (38). Therefore, we understand that the presence of two intact X chromosomes is indigence vital for normal ovarian function (8, 39-41) and prevents follicle apoptosis and atresia (41). This hypothesis is supportive for the POF etiology when there is a significant difference between the highest and lowest follicle numbers in mosaic Turner syndrome and subjects with 45, X karyotype (42).

#### Trisomy X with or without Turner’s syndrome

Chromosome aneuploidy leading to trisomy X is known as one of the genetic reasons of POF which causes elevated endocrine gonadotropine hormone (FSH) with an incidence rate of 1:1000 female live births. Although the ovarian function is normal in most of trisomy X patients, but the ovarian dysfunction in some 47, XXX might manifest as early menopause, secondary amenorrhea and oligomenorrhea (36).

Mosaic types of the syndrome include 10% of the cases with various karyotypes such as 46, XX/47, XXX or 45, X/47, XXX. The mosaic trisomy X might be the result of a post-zygotic nondisjunction event or post-zygotic trisomy rescue. The manifestation of symptoms depends on the time at which the causing events occurred.

The cytogenetic analysis done on POF patients indicated that trisomy X (regardless of mosaic or non-mosaic) have low frequency in individuals with POF (30, 37, 43).

The patients carried different symptoms with abnormalities in genitourinary tract which could be associated with the trisomy status (44). Although some of the cases showed uterine dysgenesis, other cases had no defects in the reproductive system and sexual development (37).

Investigations have shown that the autoimmune thyroid disease is related to many POF cases with trisomy X (45). The ovarian failure in 47, XXX patients (either mosaic or non-mosiac) could be the result of meiotic disorganization of three X chromosomes (20). However, more studies on 47, XXX patients with POF are required.

#### POF and gene mutations 

Numerous studies have identified different genes whose functions were significant in ovarian development and also play a role in POF progression. However, inconsistent results have been observed in different studies which are presumably the result of genetic variability between studied ethnic groups (46-48). Table 1 describes several candidate genes with possible involvement in ovarian function and POF genesis. All the introduced genic variation as mutations and polymorphisms are thought to affect POF (30). 

The FMR1 premutation of CGG repeats with incidence of 1:800 in males and 1:100-200 in women is recognized as the most important gene associated with POF (49-51). The authors mention that carrier women of the premutation are predisposed to POF disease. Expression study on fragile X mental retardation protein has demonstrated that the variation in the level of the gene product (FMRP) could be utilized as a candidate biomarker to evaluate a person with folliculogenesis disruption, heavy follicle atresia and eventual POF. In this study immunocytochemistry was applied on ovarian sections to show different FMRP1 signals in tissues with different CGG repeats. These expression studies showed that the elevated amount of the expressed gene in fetus germ line cells have a negative effect on the number of oocytes and their development (52, 53). The incidence of the disease is about 0.1-1% in normal individuals but 20-28% in the carriers of premutation FMR1, 13 times more than controls, respectively (54-58). Some studies indicated that the intermediate alleles of FMR1 CGG repeats could also increase the risk of POF development (57, 59-66).

 SF1, a nuclear receptor which is expressed in various cell types in fetus and adult, regulates different genes involved in development of the reproductive system, hypothalamic-pituitary-steroidogenesis and familial or isolated POF. The gene polymorphism Gly146Ala resulted from GGG to GCG sequence is known to be associated with POF in either familial or isolated form. Carriers of the 146Ala allele showed a significant decline in plasma stradiol. Therefore, this polymorphism could be a risk marker for POF is some women (67-72).

**Table 1:** Some candidate genes with positive influence on ovarian development and function


Gene	Chromosomelocation	The function of gene in association with POF	Reference

**FMR1**	Xq27.3	Oocyte development and number of oocytes	Allen et al., 2007 [53]
**SF1**	11q13	Regulation of gonadal sexual differentiation, follicular maturation and regulation of ovarian steroidogenesis	Ikeda et al., 1994; Leers-Sucheta et al., 1997; Luo et al., 1994; Jeyasuria et al., 2004; Reinhart et al., 1999; Lakhal et al., 2012 [67-72]
**INHA(Inhibin-alpha)**	2q33-36	Folliculogenesis	Shelling et al., 2000; Chand et al., 2010 [73-74]
**LHR (Luteinizing hormone receptor)**	2p21	Follicular growth and oocyte maturation	Pakarainen et al., 2005 [78]
**FSHR(Follicle stimulating hormone receptor)**	2p21	Follicular development	Ohkubo et al., 2013; Wei et al., 2013 [82-83]
**FOXL2**	3q23	Ovarian follicle development	Uhlenhaut and Treier, 2006; Pisarska et al., 2004; Schmidt et al., 2004; Mu et al., 2013 [89-92]
**FOXO3a**	6q21	Regulatory role in follicular activation	Watkins et al. 2006 [98]
**ER(Estrogen Receptor)**	6q25	Regulation of folliculogenesis	Kolibianakis et al. 2005 [100]
**CYP19A1**	15q21.1	Regulation of folliculogenesis through epistatic interaction with ESR1 gene; Ovary differentiation	Kim et al., 2011 Duffy et al. 2010; Kohno et al. 2010 [108-110]
**CXCL12(Chemokine)**	10q11.1	Primordial germ cell migration, colonization and survival, primordial to primary follicle transition	Doitsidou et al. 2002; Knaut et al. 2003; Molyneauxet al. 2003; Stebler et al. 2004; Herpin et al. 2008 [111-115]der001
**FMR2**	Xq28	Unknown	Murray et al. 1999 [118]
**NOBOX**	7q25	Early folliculogenesis	Rajkovic et al. 2004 [119]
**DIAPH2**	Xq22	The ovarian follicular development	Bione et al. 1998; Mandon-Pépin et al. 2003 [124-125]
**MTHFR (Methylenetetrahydrofolate reductase)**		Folliculogenesis	Laanpere et al. 2010 [127]
**LAMC1( laminin gamma 1 gene)**	1q31	High expression during ovulation	Pyun et al. 2012 [132]


Inhibins are of other POF candidate genes predominantly produced in the ovary at different times of the menstrual cycle and play a regulatory role in folliculogenesis (73, 74). Among these proteins, Inhibin alpha (INHA) gene polymorphisms have been shown to have a significant association with risk of POF in certain ethnic populations (74-77).

The luteinizing hormone (LH) through luteiniz- ing hormone receptor (LHR) plays an important role in the follicular growth and oocyte maturation. Women carrying LHR mutations showed anovula- tion and primary amenorrhea (78-81).

FSH receptor (FSHR) is expressed in the granulosa cells of the ovary and has an important function in follicular development (82, 83). In different studies on Chinese, Argentinian and British women, it has been revealed that FSH receptor gene mutations are seldom identified in POF patients. First, mutations of FSHR and later sentences are talking about them FSHR gene polymorphisms which are different terms (84-88).

FOXL2 was found in undifferentiated granulosa cells of the ovary which are involved in ovarian follicle development (89-92). The studies have indicated that FOXL2 mutations are rarely associated with POF disease (93-95).

The FOX3a is also expressed in the ovary with a regulatory role in the follicular activation (96). Although some mutations of the encoding gene have been reported in women with POF, it seems that FOX3a mutations could not be counted as a common cause (97-99).

Estrogen stimulates gonadotropins releasing at the hypothalamus-hypophysis-ovarian axis by acting on estrogen receptor-α (ESR1) which enhances folliculogenesis (100). It has been reported that several single nucleotide polymorphisms (SNPs) such as rs2234693, rs9340799 and rs2234693 of ESR1 are associated with the increased risk of POF (101-104). Some studies have suggested an association between Estrogen receptor alpha gene polymorphism, PvuII and XbaI restriction fragment length polymorphisms (RFLPs) and low bone mineral density (BMD) (105), while others observed no significant correlation between these polymorphisms with age, menopausal status and BMD (106). Nevertheless, it has been revealed that baseline BMD and change in menstrual status contributed more to the magnitude of the difference in bone change (107).

 The CYP19A1 gene encodes aromatase, the key enzyme in biosynthesis of estrogens. High expression of aromatase during the ovary differentiation has been reported previously (108, 109). Investigations on Korean patients with POF have shown a significant association between 3’ UTR SNPs "rs10046" and "rs4646" of *CYP19A1* with the disease (110).

 It has been demonstrated that *CXCL12* through its receptor acts on migration and survival of primordial germ cells (PGCs) (111-115). The association between *CXCL12* polymorphisms and POF has been observed in the studied populations (116, 117).

The *FMR2* gene is another candidate gene for POF manifestation. Microdeletions within *FMR2* have been found in some individuals with POF disease. However, the function of *FMR2* in oocyte development is still unclear (30, 118).

The newborn ovary homeobox gene (*NOBOX*) functions as an oocyte-specific gene in early folliculogenesis (119). However, the mutation analysis of *NOBOX* indicated that *NOBOX* mutations are an uncommon cause of POF (120-122).

In some POF patients, deletions within *DIAPH2* have been found along with the breakpoints at Xq22 (123). Researchers believe that the human *DIAPH2* influences the ovarian follicular development (124, 125) and that the gene is a potential candidate for POF manifestation (126).

 The variants of methylenetetrahydrofolate reductase (*MTHFR*) gene are evidenced to be associated with folliculogenesis (127). The *MTHFR* C677T and A1298C polymorphisms are significantly associated with the elevated risk of POF in different studied populations (128, 129).

Laminin is one of the most abundant components of the basal lamina. It has been demonstrated that the *LAMC1* expression increases during follicular development (130, 131). *LAMC1* variations presented a significantly association with susceptibility to POF (132).

Other genetic variations associated to POF in addition to the discussed genes in table 1, is much evidence confirming the involvement of other genes coding for small RNAs (like miRNA) during folliculogenesis (133-137). The exact function of these miRNAs is not clear yet, but germ cell development and maturation is influenced by several small RNAs such as piRNA, and siRNA that are supposed to be effective in oocyte maturation (138). The experiments indicated that the mutations or alterations of the involved genes in miRNA processing, biosynthe- sis or miRNA targets could result in increased susceptibility to sex reversal or infertility (139- 143). This effectiveness depends on the gonad- otropin hormone surge (like LH and FSH) and the pathway where a gene is able to diminish or elevate another specific activity of a gene in a certain developmental time and in a specific gonad or specific tissue during embryogenesis or ovarian development (144, 145).

In the recent years, copy number variation array (CNV array) has been an effective tool to assess the numerical variation (micro-deletion and micro-duplication) of important genes in early menopause (146). It is believed that chromosomal alterations could negatively affect germ cell apoptosis through meiotic DNA repair disruption (147).

SNPs are genetic variations which in interaction with other genes are thought to increase the risk for premature ovarian failure (148). The association data obtained from analysis of *TGFR3*, HSD17B4, *LAMC1*, ESR1, HK3 and BRSK1 are of the recent studies explaining how gene variants could be correlated to the etiology of premature ovarian failure (117, 132, 149).

#### Diagnosis and treatment

 A woman is diagnosed for POF if she has lost her regular menstrual periods for at least 4 months before the age 40. The reduction of antral follicle in POF patients could be examined by Pelvic ultrasonography (150). A new diagnostic method is the measurement of anti- Mullerian hormone (AMH) produced by antral follicles. The AMH secretion is decreased in POF patients (151, 152). The measurement of anti-adrenal, anti-ovarian and anti-thyroid autoantibodies could be useful in the diagnosis of the immune system deficiency leading to POF (150, 153). After confirming the diagnosis of POF, karyotyping and analysis of FMR1 premutation should be done to exclude major genetic causes (150). The hormone replacement therapy (HRT) is the accepted management for POF patients. Estrogen replacement is recommended to decrease the risk of osteoporosis and cardiovascular disease (154). Anxiety and depression increases in women with POF, thus, psychological support could be useful in the management of the disease (155, 156). POF is associated with complete follicular depletion and infertility. Infertility in patients with POF could be resolved by ovum donation (157). Recent studies have focused on stem cell therapy of POF. One of these investigations used CD44+/CD105+ HuAFCs (human amniotic fluid cells) to treat POF in mice and demonstrated that the cells were valid candidates for stem cell transplantation of POF due to their long half-life *in vitro* and mesenchymal potential (158).

## Conclusion

Premature ovarian failure is a complicated disorder which inhibits women’s fertility potential years before normal menopause. Most of the cases are idiopathic. Genetic variation, aberrant interaction between genes, autoimmune ovarian atrophy, iatrogenic factors, radiotherapy or chemotherapy, various environmental factors like viruses, toxins and smoking are recognized as the important agents affecting POF.

Women may encounter POF from the time of menarche and before having babies to the final years of their 30s. Many genes are found to be associated with the development, formation and function of the female reproductive system. Polymorphisms of these genes are likely to be used for the diagnosis of POF in women with normal karyotypes. What could be useful for screening and early diagnosis of mutations in these genes is the study of the association of gene polymorphisms in a large population of patients and a deeper scan of the genome including entire exons, introns and regulatory upstream and downstream regions, 5'UTR and 3'UTR regions.

Fortunately, it is easy to collect a large number of patients for testing candidate genes due to the considerable frequency of POF among fertile women (1%). Technology for mutation screening is also improving rapidly, and it will be feasible to screen a large set of candidate genes rapidly in the near future.

It is difficult to find a single candidate gene for a complex disease. Identification of the role of some important genes in POF etiology is laborious due to their involvement in several biological functions. One of these genes encodes the estrogen receptor, a nuclear factor involved in the pathogenesis of several diseases such as lung cancer, bladder cancer, osteoporosis as well as its critical role in sexual development and reproductive organization (106, 159-161). Certainly, screening a group of the genes involved in creating POF becomes easier in the future. Genetic analysis through genome-wide tests using microarray technology may identify candidate genes in patients with POF. This kind of information is helpful and informative for genetic counseling and risk assessment of POF susceptibility in family members of a particular patient.

There are conflicting data about association between some gene variants with POF, suggesting the effectivness of interactions between haplotypes of different genes on the disease etiology (75, 76, 162). A variety of analytical tools such as genome-wide association study (GWAS) could be used to find genetic variations associated with the disease (163). Another well-known and useful method is linkage analysis which can find the defective chromosomal haplotype associated with POF etiology of nonsyndromic POF (164).

It is helpful to screen women at risk for POF in early age to preserve their fertility with newly available technologies. Based on the present information, the study of X chromosome abnormalities is the easiest way to look at the immediate genetic cause of POF. Surely, after the identification of POF associated genes in the future, easier and cheaper genetic tests for early diagnosis of POF will improve the livelihood of women.
